# Jejunal diverticulosis presenting as intestinal obstruction—A case report of a rare association

**DOI:** 10.1002/ccr3.7033

**Published:** 2023-03-02

**Authors:** Munema khan, Ruqia Arshad, Irfan Malik, Ali Kamran, Fahad Gul, Ka Yiu Lee

**Affiliations:** ^1^ Department of General Surgery District Headquarter Hospital Rawalpindi Pakistan; ^2^ Department of General surgery Holy Family Hospital Rawalpindi Pakistan; ^3^ Department of Health Sciences Mid Sweden University Ostersund Sweden

**Keywords:** intestinal obstruction, jejunal diverticula, laparotomy, surgery

## Abstract

Jejunal diverticula are rare medical conditions with an incidence of 0.3%–2.5%, mostly discovered perioperatively. Our patient, 60 years old female, presented in an emergency with complaints of constipation, vomiting, abdominal pain, and distension. On examination, her abdomen was markedly distended with generalized tenderness. An erect abdominal X‐ray revealed multiple air‐fluid levels, which suggests small bowel obstruction. A diagnosis of jejunal diverticula was made on exploratory laparotomy. No evidence of granuloma or malignancy was seen on histopathological examination. Segmental resection of the affected jejunum was carried out, followed by end‐to‐end primary anastomosis. The patient was discharged on postoperative Day 6 with complete recovery at 2 weeks follow‐up visit.

## Abstract 2

Jejunal diverticulosis can cause abdominal pain, but the association with intestinal obstruction is less commonly reported. This case report highlights the need for healthcare providers to be aware of this rare association and to consider the possibility of jejunal diverticulosis in the differential diagnosis of patients with symptoms of intestinal obstruction.

## 1 INTRODUCTION

Jejunal diverticulosis is a rare condition, with an estimated prevalence of 0.1% in the general population.^1^ It is characterized by the presence of small, pouch‐like protrusions called diverticula in the jejunum, which is the middle section of the small intestine. Jejunal diverticula are usually asymptomatic and are often discovered incidentally during imaging studies or surgery for unrelated conditions.^2^ However, in rare cases, jejunal diverticulosis can present with complications such as diverticulitis, perforation, and obstruction.^3^, ^4^

Intestinal obstruction is a serious condition that occurs when the normal flow of contents through the intestine is impaired, leading to abdominal pain, nausea, vomiting, and constipation. It can be caused by various factors such as mechanical blockage, inflammation, or cancer.^5^ Jejunal diverticulosis presenting as an intestinal obstruction is an extremely rare association, with only a few cases reported in the literature.^6^, ^7^

The management of jejunal diverticulosis presenting as intestinal obstruction can be challenging as it requires a thorough understanding of the underlying pathophysiology and appropriate selection of treatment modalities.^8^

Due to delayed diagnosis, patients often present critically and warrant operative intervention, conventionally a segmental resection. Recently, a trend towards conservative management is being increasingly reported to have favorable outcomes. Hence, high index of suspicion and a novel approach to diagnostic deduction are key when confronted with such patients. A timely diagnosis can save the patient from unnecessary exploration.

We are presenting the case of a 50‐year‐old female patient who presented to us with complete small bowel obstruction. The novelty of this case report lies in the rare association between jejunal diverticulosis and intestinal obstruction. While it is well known that diverticulosis can cause abdominal pain and other symptoms, the association with intestinal obstruction is less commonly reported. This case report highlights the need for healthcare providers to be aware of this rare association and to consider the possibility of jejunal diverticulosis in the differential diagnosis of patients with symptoms of intestinal obstruction.

## 2 CASE PRESENTATION

A 60‐year‐old normotensive, normoglycemic female patient was referred to our emergency department with complaints of constipation for 10 days, vomiting for 3 days, and abdominal pain and distension for 1 day. There was no relevant family or drug history. The pain and distension were worsening in the last 24 h. There were no aggravating or relieving factors. Her vital signs were slightly abnormal with a pulse rate of 92 beats per min, blood pressure of 112/70 mmHg, and a body temperature of 98.6. On abdominal examination, her abdomen was markedly distended with generalized tenderness and her bowel sounds were slightly sluggish on auscultation.

**FIGURE 1:**
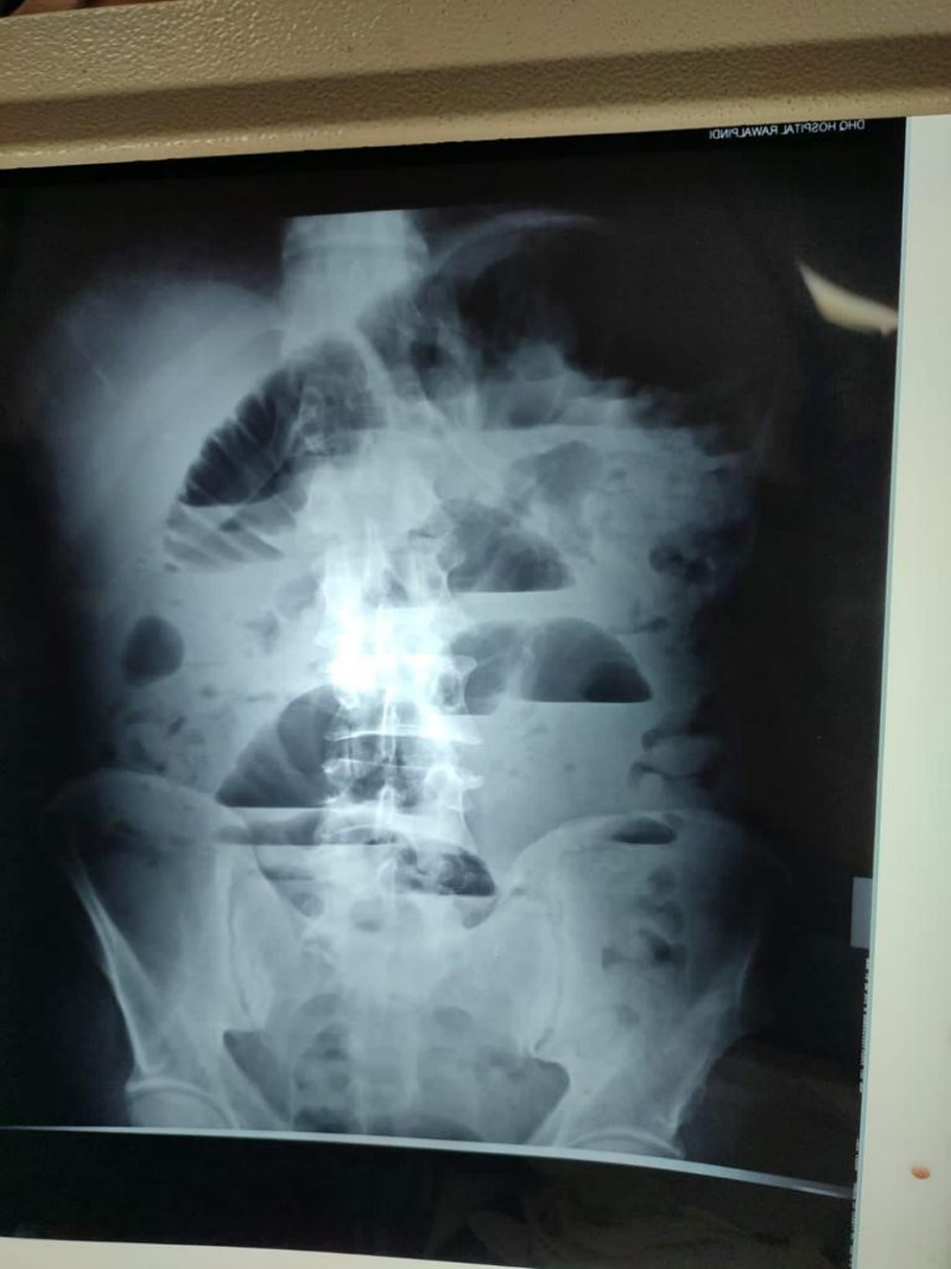
Erect abdominal X‐ray with multiple fluid levels.

Baseline blood work showed leukocyte count 8.2 10^3u/L, serum amylase 96u/L with remaining parameters in the normal range. Her radiological investigations were carried out and erect abdominal X‐rays revealed multiple air‐fluid levels suggesting intestinal obstruction in the small bowel (Figure 1).

**FIGURE 2:**
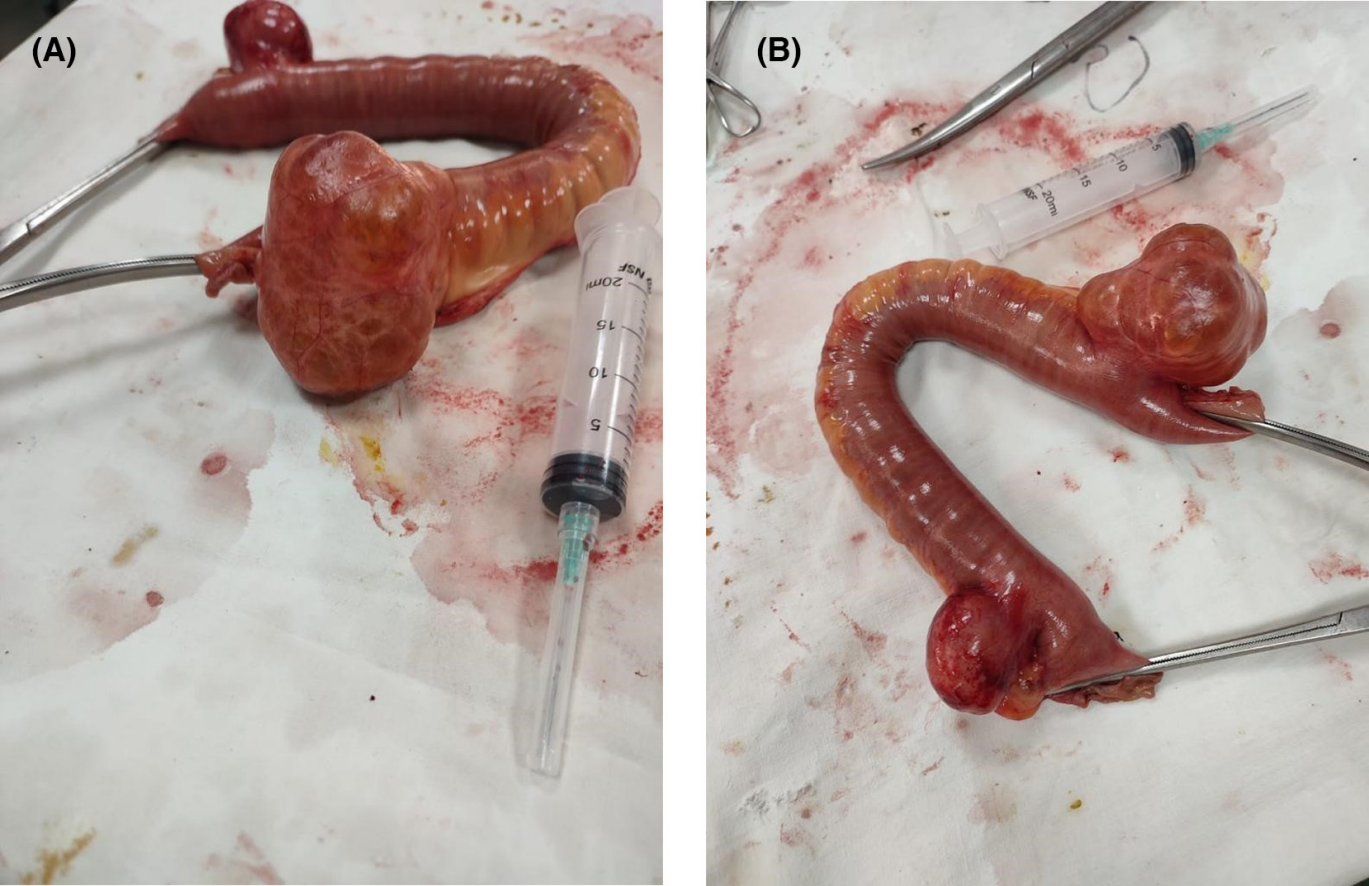
Jejunal diverticula.

The patient underwent exploratory laparotomy (by SR and residents) which macroscopically revealed the diagnosis of jejunal diverticula. Two jejunal diverticula at 1 and 2 feet from the DJ were present, and the second diverticula was adherent at the ileocecal junction, causing intestinal obstruction. A segmental (20 cm) resection of the affected jejunum was carried out, followed by an end‐to‐end hand‐sewn primary anastomosis (Figure 2). Postoperatively, a 5‐day course of antibiotics was administered. The patient was able to tolerate oral intake after the removal of the nasogastric tube. The patient was discharged home on Day 6. At the two‐week follow‐up visit, she fully recovered. No adverse or unanticipated events were registered 3 months later.

Histopathological examination confirmed the diagnosis and showed a small bowel lined by intact epithelium. Sections from the diverticula showed a moderately dense inflammatory infiltrate composed of lymphocytes, plasma cells, neutrophils, eosinophils, and histiocytes. No evidence of granuloma or malignancy was seen.

## 3 DISCUSSION

Small Bowel Diverticula are a rare and unusual entity and are significantly less prevalent than their colonic counterparts.^2^, ^6^ They can be acquired or congenital, true, or false but except for Meckel's diverticulum, all are considered false, acquired pulsion diverticula. Mostly found in the duodenum, the incidence in the jejunum or ileum, termed jejunoileal diverticula (JID) is only 18%. Inversely in contrast to duodenal diverticula, jejunal diverticula have an 18‐fold chance of perforation.^3^ Pre‐operative diagnostic yield is lacking given the lack of clinical familiarity and asymptomatic pathological profile. Fatal complications can result and the incumbent delayed diagnosis further adversely impacts the clinical course.^7^ If detected early, pre‐op characterization can justify a conservative course of treatment, which is especially important in the patient cohort in which the pathology manifests.^2^

Jejunal diverticula are mostly multiple and occur predominantly in older men in the sixth or seventh decade.^1^, ^3^ No clear etiology has yet been established.^3^ They are linked to connective tissue disorders, systemic sclerosis, visceral myopathies, and neuropathies.^1^, ^8^ The most widely held theory entails that JID is pulsion diverticula resulting from intestinal dysmotility.^1^ Increased intraluminal segmental pressure exploits the weak points in the muscularis mucosae with resultant herniation of the mucosa and sub‐mucosa.^6^, ^9^ The weak pathological points are the entry of vasa recta from the jejunal arcades, and hence, almost all cases on review are found on the mesenteric border, often obscured by the mesenteric fat.^10^ Size is variable ranging from a few mm up to 10 cm.^7^ Only 4–5 cases on the antimesenteric site are found in the literature review.^6^, ^7^, ^9^, ^11^ True diverticula are scantily reported but theorized to carry a better prognosis attributed to the increased durability of their wall.^12^ Thus, heterogeneous pathophysiology is highlighted mandating further probing and deliberation on the subject.

Mostly asymptomatic, JD is discovered at autopsy or preoperatively for unrelated surgery and increasingly during imaging studies. When symptomatic, the presentation can either be vague or it can be life‐threatening in case of ensuing complications. Mostly they tend to mimic colonic diverticulitis.^13^ Complications occur in 6%–10% of cases.^6^, ^10^ Notably, a review states that while jejunal diverticula had an incidence of 18%, they were associated with 46% of complications.^1^ Typically, bacterial overgrowth causes malnutrition and symptoms of dyspepsia and upper abdominal pain which are often dismissed.^2^, ^3^ Complications can range from common such as diverticulitis, obstruction, perforation, and hemorrhage^1^, ^3^, ^7^ to Rare, that is, neoplasia, GIST, and arteriovenous malformation.^4^ Nobel et al suggested a triad of obscure abdominal pain, anemia, and dilated small bowel loops on abdominal radiographs for diagnosis.^14^ Obstruction when present is mostly due to an enterolith and hemorrhage can be massive enough to invoke massive transfusion protocols.^7^

Obstruction can also be caused by intussusception or by extrinsic compression from a nearby loop containing a diverticulum^7^ or by adhesion bands or strictures from associated diverticulitis as in our case.^4^ Enterolith formation is aided by the acidic environment within the diverticula.^7^ In our case, underlying diverticulitis was found with a resultant adhesion band causing entrapment of the bowel. Diverticulitis is traditionally responsive to conservative therapy^2^ and while it has been reported, it is seldom accompanied by complete obstruction^2^ Mirroring our case but differing in the therapeutic approach, Elfanagley et al and Lin CH et al^4^ report the obstruction settling with conservative therapy but ultimately to prevent recurrence of subacute intestinal obstruction, elective resection was opted.^6^

Enteroscopy and enteroclysis are the most sensitive and specific diagnostic tools in hemodynamically stable patients.^2^, ^14^, ^15^ Their availability remains mutable and subject to resources and emergent nature of the presentation; they are often precluded. CT is clinically useful, its utility being its ability to be employed both diagnostically as well as therapeutically. In the former capacity, it can categorize the severity of the disease and exclude complications. In the latter, it can enable percutaneous drainage.^13^ Thus in many cases, CT is playing a decisive role, in shaping the treatment algorithm.^5^ Endoscopy and magnetic resonance enterography/enteroclysis (MRE) are now also added to the diagnostic armamentarium reportedly replacing enteroclysis with highly specific features reported by Mansoori et al.^16^

In asymptomatic JID no intervention is required and when mandated, it is tailored to the presenting complication.^1^, ^4^, ^8^ Previously segmental resection and re‐anastomosis were the established convention/gold standard but a shift favoring conservative treatment of Diverticular perforation/complications with anti‐biotherapy and percutaneous drainage is now being widely reported and recommended.^1^, ^2^, ^10^, ^17^ Inclusive of this is the exclusion of diffuse peritonitis and Pneumoperitoneum^3^, ^10^ contrasting school of thought differs and recommends pre‐emptive intervention given possible complications.^6^ Gastrointestinal hemorrhage is amenable to angiography and angioembolization before appropriate resection. Enterectomy is the gold standard approach with Diverticulectomy not preferred due to the high risk of anastomotic leak.^3^, ^8^, ^14^ In the case of multiple diverticula, all are segmentally resected. For an enterolith, simple enterotomy or manual crushing of the stone and milking to ICJ can be done as per convention.^14^

In our case, we opted for enterectomy and anastomosis consistent with the approach widely reported.^1^, ^3^, ^6^, ^10^ All patients had good postoperative outcomes similar to ours.

## 4 CONCLUSION

JID is a rare and intriguing variation of the normal small bowel pathology. They warrant attention not just on account of the potentially fatal clinical implications but also based on the pathophysiological enigma that they pose. Surgeons must be adequately primed to be alert to the presence of this entity as often a conservative approach can be steered at rather than a physiologically disrupting invasive exploration.

## AUTHOR CONTRIBUTIONS

**Munema Khan:** Investigation; methodology; writing – original draft. **Ruqia Arshad:** Investigation; methodology; writing – review and editing. **Irfan Malik:** Investigation; methodology; writing – review and editing. **Ali Kamran:** Investigation; methodology; writing – review and editing. **Fahad Gul:** Investigation; methodology; writing – review and editing. **Ka Yiu Lee:** Investigation; methodology; writing – review and editing.

## FUNDING INFORMATION

None.

## CONFLICT OF INTEREST STATEMENT

None.

## ETHICS STATEMENT

Ethical approval was not required as per country guidelines.

## CONSENT

Written informed consent was obtained from the patient to publish this report in accordance with the journal's patient consent policy.

## Data Availability

Data are available from the first author (Munema Khan) upon reasonable request.
